# Advancing understanding of executive function impairments and psychopathology: bridging the gap between clinical and cognitive approaches

**DOI:** 10.3389/fpsyg.2015.00328

**Published:** 2015-03-26

**Authors:** Hannah R. Snyder, Akira Miyake, Benjamin L. Hankin

**Affiliations:** ^1^Department of Psychology, University of Denver, DenverCO, USA; ^2^Department of Psychology and Neuroscience, University of Colorado Boulder, BoulderCO, USA

**Keywords:** psychopathology, executive function, inhibition, shifting, working memory, methods, transdiagnostic

## Abstract

Executive function (EF) is essential for successfully navigating nearly all of our daily activities. Of critical importance for clinical psychological science, EF impairments are associated with most forms of psychopathology. However, despite the proliferation of research on EF in clinical populations, with notable exceptions clinical and cognitive approaches to EF have remained largely independent, leading to failures to apply theoretical and methodological advances in one field to the other field and hindering progress. First, we review the current state of knowledge of EF impairments associated with psychopathology and limitations to the previous research in light of recent advances in understanding and measuring EF. Next, we offer concrete suggestions for improving EF assessment. Last, we suggest future directions, including integrating modern models of EF with state of the art, hierarchical models of dimensional psychopathology as well as translational implications of EF-informed research on clinical science.

## Introduction

Executive function (EF) is essential for successfully navigating nearly all of our daily activities. EF is comprised of a set of cognitive control processes, mainly supported by the prefrontal cortex (PFC), which regulate lower level processes (e.g., perception, motor responses) and thereby enable self-regulation and self-directed behavior toward a goal, allowing us to break out of habits, make decisions and evaluate risks, plan for the future, prioritize and sequence our actions, and cope with novel situations (e.g., Banich, 2009; Miyake and Friedman, 2012). Individual differences in EF are associated with many important aspects of human health and functioning, including academic and occupational functioning (e.g., Best et al., 2009; Miller et al., 2012b; Valiente et al., 2013), interpersonal problems (e.g., Sprague et al., 2011; De Panfilis et al., 2013), substance use (e.g., Nigg et al., 2006; Ersche et al., 2012), physical health (e.g., Hall et al., 2006; Falkowski et al., 2014), and mental health (e.g., Willcutt et al., 2005; Bora et al., 2009; Mesholam-Gately et al., 2009; Snyder, 2013).

Of critical importance for clinical psychological science, EF impairments are associated with most forms of psychopathology, as discussed below. Moreover, poor EF predicts rumination (e.g., Whitmer and Banich, 2007; De Lissnyder et al., 2012; Demeyer et al., 2012; Zetsche et al., 2012), worry (Crowe et al., 2007; Snyder et al., 2010, 2014) and poor use of adaptive emotion regulation strategies (e.g., reappraisal, McRae et al., 2012; Andreotti et al., 2013), which are all potent risk factors for multiple forms of psychopathology (e.g., Ruscio et al., 2007; Aldao et al., 2010; Abela and Hankin, 2011; McLaughlin and Nolen-Hoeksema, 2011). Thus, it has been proposed that EF deficits may be transdiagnostic intermediate phenotypes or risk factors for emotional, behavioral, and psychotic disorders (e.g., Nolen-Hoeksema and Watkins, 2011; Buckholtz and Meyer-Lindenberg, 2012; Goschke, 2014).

However, despite the proliferation of research on EF in clinical populations, the history of cognitive approaches in psychopathology has followed a curious path, best illustrated as mostly parallel play, between two predominantly independent scientific traditions: clinical psychology/psychiatry and cognitive psychology/cognitive neuroscience. With notable exceptions, this theme of parallel play between clinical and cognitive science is largely reflected up to the present, and sometimes leads to failures to apply theoretical and methodological advances in one field to the other field, hindering progress.

This paper has three main goals. First, we review the current state of knowledge of EF impairments associated with psychopathology and limitations to the previous research in light of recent advances in understanding and measuring EF. Specifically, while EF impairments appear to be transdiagnostically associated with psychopathology, conceptual and methodological limitations of prior research make existing evidence difficult to interpret–thus, the specific nature and pattern of EF impairments, both across different aspects of EF and across forms of psychopathology, remains unclear. We argue that investigating how specific aspects of psychopathology affect, and are affected by, specific aspects of EF is critical for pushing clinical psychological science forward toward beginning to understand the underlying cognitive, neural, and genetic mechanisms involved at a level that will enable translational research to improve interventions. Next, we offer concrete suggestions for improving assessment of EF, based on both conceptual and methodological issues in current research practices with EF, to advance clinical psychological science. We advocate for better assessment of EF using the best current, validated models of EF and best methods for assessing EF. Specifically, we provide recommendations for applying validated models of EF to clinical research, using multiple tasks to obtain purer measures of EF, and selecting and analyzing tasks in ways that minimize the inherent noisiness of EF data. Last, we suggest future directions in research with EF and clinical psychological science, including integrating modern models of EF with state of the art, hierarchical models of dimensional psychopathology as well as translational implications of EF-informed research on clinical science.

## EF Impairments Associated with Psychopathology: Current State of Knowledge

Executive function is best characterized as consisting of separable but related cognitive processes, with both unique and shared individual differences, genetic influences, and neural substrates (e.g., Miyake and Friedman, 2012), a topic we return to in the following section. **Table 1** defines the aspects of EF that have been most heavily studied in clinical populations, including shifting, inhibition, updating, and working memory (WM). Importantly, many of these components can be further subdivided. For example, manipulating information in WM places heavier demands on EF (i.e., the *central executive* component of WM) than simple maintenance (e.g., Baddeley and Repovs, 2006). WM maintenance can further be divided into verbal (e.g., words, letters and numbers) and visuospatial (e.g., shapes, patterns and spatial locations), while the central executive component of WM is believed to be domain-general (e.g., Baddeley and Repovs, 2006). It should be noted that in the literature, terms such as *attentional control* and *executive attention* are sometimes used to refer to the same tasks and cognitive processes referred to elsewhere as EF. This is largely a matter of differences in terminology rather than substance, as there is often agreement between those employing different terminology on the underlying cognitive and neural mechanisms (e.g., Petersen and Posner, 2012). We further elaborate on EF constructs in Conceptual Models below.

**Table 1:** Executive function (EF) processes and measures.

EF process	Definition	Traditional neuropsychological measures	More specific EF measures
**Shifting**	Switching between task sets or response rules (e.g., you may need to shift from reading this paper to responding to an urgent email and back again)	**Wisconsin card sorting task (WCST):** Learn from feedback to sort cards by one dimension (e.g., color), and then switch to a different dimension (e.g., shape) when given negative feedback on the first dimension (repeats with multiple sorting rules). Common measures: perseverative errors, number of switches achieved.	**Category Switch:** Perform one of two tasks (categorize by size or by living vs. non-living) depending on cue before each trail. Common measures: switch cost (switch – repeat trial RT) For methods see: Friedman et al. (2008)
		**Trail making B:** Alternately connect letters and numbers in sequence (A-1-B-2 etc.). Often compared to trail making A (connect letters or numbers only, does not require shifting). Common measures: time to complete B, TMT-B – TMT-A completion time.	**Number-letter switch:** Perform one of two tasks (categorize number-letter pairs by odd/even number or vowel/consonant) depending on cue before each trail. Common measures: switch cost (switch – repeat trial RT) For methods see: Friedman et al. (2008)
		**Object alternation test (OAT)/delayed alternation test (DAT):** find object hidden alternately in two locations, with or without a delay before being allowed to search. Common measures: errors	**Color-shape switch:** Perform one of two tasks (categorize colored shapes by shape or color) depending on cue before each trail. Common measures: switch cost (switch – repeat trial RT) For methods see: Friedman et al. (2008)
			**CANTAB intradimensional/extradimensional shift (ID/ED):** Learn from feedback to select a stimulus based on one dimension, switch to the previously non-rewarded stimulus (intradimensional shift), then to a different stimulus dimension (extradimensional shift). Common measures: perseverative errors, number of switches achieved, time to completion. For methods see: Robbins et al. (1998); Commercially available (http://www.cambridgecognition.com)
**Inhibition**	Suppressing or resisting a prepotent (automatic) response in order to make a less automatic but task-relevant response (e.g, you may want to resist the automatic response of checking those not-so-urgent emails in order to complete reading this paper.	**Color-word Stroop (neuropsychological version):** Separate blocks of word reading, color naming, and incongruent (e.g., “red” written in blue ink) trials. Common measures: incongruent block time, incongruent – color naming block time, incongruent block errors.	**Color-word Stroop (experimental version):** Identify the color ink a color word is printed in. Trials are incongruent (e.g., “red” written in blue ink) and congruent (e.g., “red” written in red ink) or neutral (non-color word or asterisks) trail RTs. Trial types are randomly intermixed. Common measures: interference (incongruent – neutral RT/neutral RT), incongruent – neutral errors For methods see: Friedman et al. (2008)
		**Go/no-go:** Quickly categorize and respond to some stimuli, and withhold a response to other stimuli. Common measures: Commission (no-go) and omission (go) errors	**Stop signal:** Quickly categorize and respond to stimuli (e.g., left and right pointing arrows), unless a stop signal appears, signaling to withhold a response. Common measures: stop signal RT (SSRT, time needed to stop a response). For methods see: Verbruggen et al. (2008)
		**Hayling:** Read sentences where the final word is omitted but highly predictable. First complete sentences correctly (Part A), then with an unrelated word (part B). Common measures: Part B – Part A RT, Part B errors.	**Antisaccade:** Look in the opposite direction of visual cue to detect a briefly presented target. Common measures: errors (detected by eye tracking or failure to detect briefly presented target on correct side). For methods see: Friedman et al. (2008)
**Updating**	Monitoring and coding incoming information for task-relevance, and replacing no longer relevant information with newer, more relevant information (e.g., as you read this paper, you may be monitoring for a relevant piece of information you are looking for, hold this information in working memory while you write it down, then replace it with the next relevant piece of information.)		**Verbal *n*-back:** Indicate if the stimulus (usually letter) matches the stimulus *n* (e.g., 3) items back. Common measures: accuracy For methods see: Kane et al. (2007)
			**Spatial *n*-back:** Indicate if the spatial location of a stimulus matches the location *n* (e.g., 3) items back. Common measures: accuracy For methods see: Friedman et al. (2008)
			**Letter memory:** Remember and repeat the last three letters in a letter string, adding the most recent letter and dropping the fourth letter back. For methods see: Friedman et al. (2008)
			**Keep Track:** Remember to last exemplar word presented in several target categories and report these words at the end of the trail. Common Measures: Accuracy For methods See: Friedman et al. (2008)
**Working memory manipulation**	Actively maintaining (i.e., ‘holding on line’) and manipulating information across a short delay.	**Digit span backward:** Repeat sequence of numbers in reverse order. Common measures: span (max. correct sequence length)	**Reading span:** Read a serious of unrelated sentences, then recall the last word of each sentence. Common measures: number of words correctly recalled For methods see: Conway et al. (2005)
		**Self-ordered pointing:** Search an array of boxes for hidden tokens. Token is only in each location once. Common measures: errors (return to previous location), Strategy score (how often search is initiated from same starting box).	**Operation span:** Read aloud and verify simple math equations, then read aloud a presented word. At end of trial, recall all words. Common measures: number of words correctly recalled For methods see: Conway et al. (2005)
			**Spatial span backward:** Click irregularly arranged squares in the opposite order as they light up on the computer screen. Common measures: span, number of correct sequences. For methods see: Berch et al. (1998)
**Working memory Maintenance**	Actively maintaining (i.e., ‘holding on line’) information across a short delay, without the need to manipulate that information.	**Digit span forward:** Repeat sequence of numbers in forward order. Common measures: span (max. correct sequence length)	**Corsi block tapping/spatial span forward/ CANTAB spatial working memory:** Tap irregularly arranged blocks/squares in the same order as experimenter (Corsi blocks) or computer (spatial span). Common measures: span For methods see: Berch et al. (1998)
		**Delayed match-to-sample:** View a complex shape (the sample), then indicate after a delay if a probe matches the sample. Common measures: accuracy	
**Less specific tasks**			
Verbal Fluency		**Semantic verbal fluency/category fluency:** Say as many words from a semantic category (e.g., animals) as possible in 1 (or 3) min. Common measures: number of words	**If the goal is to assess verbal fluency:** Score verbal fluency for more specific measures: switching (transitions between subcategories) and clustering; consider using weighted switch scores to avoid confounding these two measures. **If the goal is to assess EF rather than verbal fluency *per se*:** Consider using more specific tasks above. For methods see: Abwender et al. (2001), Snyder and Munakata (2010)
		**Phonemic verbal fluency/controlled oral word association (COWA):** Say as many items starting with a certain letter (usually F, A, S) as possible in 1 (or 3) min. Common measures: number of words	
Planning		**Tower of London (TOL)/CANTAB stockings of Cambridge (SOC):** Move rings on pegs from a starting position to a target position in as few moves as possible, following a set of rules. Common measures: number of perfect solutions, number of moves in excess of minimum, completion time.	**If the goal is to assess planning:** Score for more sensitive measures (e.g., RT per move, number of moves) **If the goal is to assess EF rather than planning *per se*:** Consider using more specific tasks above.
		**Tower of Hanoi:** Move rings from one peg to another in as few moves as possible without placing a larger ring on top of a smaller one. Common Measures: Number of perfect solutions, number of moves in excess of minimum, completion time.	

Tasks listed under traditional neuropsychological measures are measures that have been extensively used to assess EF in clinical populations, but often lack specificity, and sometimes also lack sensitivity (e.g., there may be ceiling effects in less impaired populations). These tasks may be most appropriate for screening for severe EF deficits in patients. Tasks listed under more specific EF measures are designed to more specifically target aspects of EF and to be sensitive to individual differences in EF across a wider range of abilities. These tasks are more appropriate for studies probing the nature of more subtle EF deficits associated with psychopathology.

Only tasks with emotionally neutral materials (i.e., “cold” EF tasks) were included in the current review. This was done to avoid confounding altered emotional processing with EF impairments. That is, impairments on tasks involving affective or disorder-related materials could arise from either impairments in EF processes or increased salience of these materials, and thus distractibility, for individuals with psychopathology, making results difficult to interpret. However, it should be noted that impairments on “hot” EF tasks are also present across many disorders, and in some cases may be larger than those on “cold” EF tasks, especially when disorder-specific materials are used (for reviews, see Williams et al., 1996; Bar-Haim et al., 2007; Peckham et al., 2010; Cisler et al., 2011).

The previous research on EF impairments associated with psychopathology reviewed in this section has primarily used cross-sectional (case-control) designs in adult samples, and assessed EF with the traditional neuropsychological tasks in the third column of **Table 1**. Despite considerable knowledge, as summarized in **Table 2** and below, there are several important limitations inherent in the primary literature. These methodological and conceptual issues impose constraints on the state of knowledge and what can be determined through meta-analysis. First, many neuropsychological EF measures tap multiple aspects of EF as well as non-EF abilities. Such traditional but non-specific tasks may be useful for screening individuals for severe EF deficits, however, they are too broad to answer fine-grained questions about specific aspects of EF and potential underlying mechanisms relating EF aspects to forms of psychopathology. For example, verbal fluency tasks have been a perennial favorite for assessing EF. However, the verbal fluency and other complex neuropsychological tests tap a wide variety of cognitive processes, including not only multiple aspects of EF (e.g., shifting between subcategories, WM for what items have already been named), but also non-executive abilities (e.g., semantic memory). Even seemingly more specific tasks, such as the Wisconsin card sorting test (WCST) require other complex cognitive processes (e.g., learning from feedback). Second, because they were developed to detect more severe deficits (e.g., due to brain damage) many traditional neuropsychological tasks may lack sensitivity to detect more subtle EF deficits. That is, in some cases effect sizes for a particular EF task may be smaller than another not because of true differences in the magnitude of impairments on different aspects of EF, but merely because one task suffers from ceiling effects.

**Table 2:** Summary of recent EF meta-analyses.

						Measures of more specific EF components							Non-Specific Measures		
	Meta-analysis	Clinical Group	*K*^1^	*N*^2^	Publication bias Correction^3^	Shifting	Inhibition		Updating	Verbal WM Manip.	Verbal WM Maint.	Visuospatial WM	Phonemic VF	Semantic VF	Planning
MDD	Rock et al. (2013)	MDD	24	1,511	No	0.44	–		–	–	–	0.45	–	–	0.43
	Snyder (2013)	MDD	113	7,707	Yes	0.47	0.58		0.57	0.52	0.39	0.45	0.46	0.70	0.38
Average						**0.46**	**0.58**		**0.57**	**0.52**	**0.39**	**0.45**	**0.46**	**0.70**	**0.41**
BD	Arts et al. (2008)	Euthymic BD (all)	28	1,028	No	0.94	0.73			1.02	0.37	–	0.59	0.87	–
	Bora et al. (2009)	Euthymic BD (all)	45	1,423	Yes	0.78	0.76			0.75	0.37	–	0.60	–	–
	Bora et al. (2011)	Euthymic BD II	9	678	Yes	0.51	0.72			–	0.39	–	0.47	0.46	0.29
	Kurtz and Gerraty (2009)	Euthymic BD (all)	42	NR	Yes	0.67	0.75			0.65	0.41	–	0.51	0.75	–
	Kurtz and Gerraty (2009)	Manic/mixed BD (all)	13	NR	Yes	0.68	–		–	–	–	–	0.51	0.59	–
	Kurtz and Gerraty (2009)	Depressed BD (all)	5	NR	Yes	0.64	–		–	–	–	–	0.93	–	–
	Mann Wrobel et al. (2011)	Euthymic BD (all)	28	2,410	Yes	0.73	0.78			0.81	0.40	0.55	0.55	0.58	–
	Robinson et al. (2006)	Euthymic BD (all)	26	1,410	Yes	0.77	0.63			0.98	0.47	–	0.34	1.09	–
	Torres et al. (2007)	Euthymic BD (all)	39	2,076	No	0.62	0.71			0.54	–	–	–	–	_
	Walshaw et al. (2010)	Pediatric BD (all)	16	777	No	0.73	0.46			–	–	0.80	0.34	0.38	0.96
Average						**0.71**	**0.69**		–	**0.79**	**0.40**	**0.68**	**0.54**	**0.67**	**0.63**
OCD	Abramovitch et al. (2013)	OCD	115	6,716	Yes	0.52^4^	0.49		–	0.34^2^		0.37^2^	–	–	0.44
	Shin et al. (2013)	OCD	88	6,094	Yes	0.42	0.55		–	0.11		0.49	0.39	0.42	0.73
	Snyder et al. (2015)	OCD	110	6,315	Yes	0.50	0.37		0.71	0.31	0.07	0.47	0.39	0.34	0.44
Average						**0.48**	**0.47**		**0.71**	**0.22**		**0.44**	**0.39**	**0.41**	**0.54**
PTSD	Polak et al. (2012)	PTSD vs. trauma exposed controls	18	1,080	No	0.70	0.10		–	0.45	–	–	–	–	–
Schizophrenia	Bokat and Goldberg (2003)	Schizophrenia	13	915	No	–	–		–	–	–	–	0.99	1.27	–
	Dickinson et al. (2007)	Schizophrenia	37	3,405	Yes	0.87	0.99		–	0.86	0.73	–	0.83	1.41	–
	Doughty and Done (2009)	Schizophrenia	91	NR	Yes	–	–		–	–	–	–	–	1.34	–
	Forbes et al. (2009)	Schizophrenia	187	NR	Yes	–	–		–	1.08	0.82	0.87	–		–
	Henry and Crawford (2005)	Schizophrenia	84	5,416	Yes	0.98	0.98		–	–	–	–	0.95	1.12	–
	Mesholam-Gately et al. (2009)	First episode psychosis	43	4,979	Yes	0.86	0.88		–	0.79	0.50	0.80	0.69	1.24	–
	Piskulic et al. (2007)	Schizophrenia	33	2,353	No	–	–		0.83	–	–	1.09	–	–	–
	Stefanopoulou et al., 2009	Schizophrenia	11	963	No	0.99	–		–	–	–	–	1.03	–	–
Average						**0.92**	**0.95**		**0.83**	**0.91**	**0.68**	**0.92**	**0.90**	**1.28**	–
ADHD							Stroop	Motor							
	Alderson et al. (2013)	ADHD adults	30	2,731	Yes					0.55^5^	–	0.49			
	Alderson et al. (2007)	ADHD children and adolescents	22	NR	Yes		–	0.63	–	–	–	–	–	–	–
	Bálint et al. (2009)	ADHD adults	25	3,442	Yes	0.72	0.30	–	–	–	–	–	–	–	–
	Boonstra et al. (2005)	ADHD adults	13	1,099	No	0.65	0.13	–	–	0.44	0.29	–	0.62	–	–
	Frazier et al. (2004)	ADHD all ages	137	5,800	Yes	0.50	0.56	0.54	–	–	–	–	0.46	0.41	–
	Hervey et al. (2004)	ADHD adults	33	2,475	No	–	0.15	–	–	–	–	–	–	–	–
	Lansbergen et al. (2007)	ADHD adults	18	1,362	No	–	0.24	–	–	–	–	–	–	–	–
	Lijffijt et al. (2005)	ADHD adults	29	2,055	No	–	–	0.58	–	–	–	–	–	–	–
	van Mourik et al. (2005)	ADHD all ages	17	2,395	No	–	0.35	–	–	–	–	–	–	–	–
	Walshaw et al. (2010)	ADHD children and adolescents	68	5,728	No	0.37	0.38	0.63	–	0.63	–	0.86	0.68	0.38	0.38
	Willcutt et al. (2005)	ADHD children and adolescents	83	6,703	No	0.51	–	0.61	–	0.55	–	0.63	–	–	0.60
Average						**0.55**	**0.30**	**0.63**	–	**0.54**	**0.29**	**0.66**	**0.59**	**0.41**	**0.49**
Substance use disorders^6^	Spronk et al. (2013)	Long-term cocaine users	63	NR	No	0.37	–	0.58	–	–	–	–	–	–	–
	Smith et al. (2014)	Cocaine users	19	942	No			0.45	–				–	–	–
	Smith et al. (2014)	Alcohol dependence	18	1,454	No	–	–	0.46	–	–	–	–	–	–	–
	Smith et al. (2014)	MDMA users	5	198	No			0.35							
	Smith et al. (2014)	Metham-phetamine users	4	178	No			0.72							
	Smith et al. (2014)	Cannabis users	11	739	No			0.06							
	Smith et al. (2014)	Tabacco users	12	595	No			0.23							

Summary of meta-analyses conducted in the last 10 years. Weighted mean effect size (Cohen’s *d*) comparing healthy control participants to the clinical group. All effect sizes have been recoded such that positive values represent worse task performance by the clinical group. When a meta-analysis reported effect sizes for multiple individual tasks within an EF component, the average of these effect sizes is reported.

^1^Number of studies included in the meta-analysis. Note that the number of studies included in effect size varies. See cited articles for details.

^2^Number of participants in the meta-analysis. Note that the number of participants included in effect size varies. See cited articles for details.

^3^Yes = Meta-analysis provided evidence for lack of publication bias and/or corrected for publication bias (e.g., trim and fill).

^4^Composite measures including some tasks we would not classify as shifting (verbal fluency, design fluency, and WAIS similarities) in addition to traditional shifting tasks.

^5^Includes updating tasks.

^6^All substance use disorder meta-analyses include studies with poly-substance use, so effects of individual drugs should be interpreted with extreme caution.

Manip., manipulation; Maint., maintenance; VF, verbal fluency; NR, not reported.

Finally, these limitations carry over into meta-analyses. Both in our own classification in **Table 2** and many of the original meta-analyses summarized in the table, tasks are grouped into the EF processes they are commonly considered to tap (e.g., WCST is classified as a shifting task). However, these categories may be lumping together tasks that may actually be tapping different, and multiple, processes. Specifically, tasks classified as tapping a particular aspect of EF may not be sensitive measures of that process, and are not pure measures of that EF process, since they often require other EF processes as well as multiple non-EF processes.

Despite these limitations, the meta-analytic evidence summarized in **Table 2** indicates that EF deficits are pervasive across disorders and EF tasks, although the magnitudes of these deficits vary. We first briefly summarize what is currently known based on this previous research, and argue that progress in understanding the nature, origins, and consequences of EF impairments associated with psychopathology will require more specific measures and better conceptual models. We then lay out some concrete suggestions for advancing research in these directions.

### Impairments on More Specific EF Components: Inhibition, Shifting, Updating, and Working Memory

The largest EF deficits are found for individuals with schizophrenia, with large effect sizes on measures of shifting, inhibition, updating, visuospatial WM, and verbal manipulation, and a medium effect size for simple verbal WM maintenance. These EF tasks are also impaired in individuals with mood disorders, although the magnitude of these deficits is somewhat smaller than those in schizophrenia. Meta-analytic evidence demonstrates that individuals with major depression (MDD) are significantly impaired, with similar small-to-medium effect sizes, on measures tapping shifting, inhibition, updating, and WM. Similarly, while individuals with bipolar disorders (BDs) have somewhat larger impairments than individuals with MDD, they are also relatively uniformly impaired across EF domains, with medium effect sizes for shifting, inhibition, visuospatial WM, and verbal WM manipulation, and a small but significant effect for verbal WM maintenance. There is little research on updating in individuals with BD. Individuals with obsessive compulsive disorder (OCD) also have impaired performance across these core EF domains, with small but significant effect sizes for shifting, inhibition, visuospatial WM, and verbal WM manipulation, but a large effect size for updating. In contrast, simple WM maintenance appears to be unimpaired in individuals with OCD. Importantly, while depression frequently co-occurs with OCD, EF deficits in OCD are not driven by co-occurring depression, as even those with low levels of depressive symptoms show the same level of EF deficits (Snyder et al., 2015).

A recent meta-analysis found that compared to trauma-exposed individuals who did not develop post traumatic stress disorder (PTSD), individuals with PTSD had worse performance on measures of shifting, with a medium effect size, and visuospatial WM, with a small effect size, but not the Stroop measure of inhibition (other EF components were not analyzed; Polak et al., 2012). However, a review of the literature including a wider range of inhibition tasks suggests that individuals with PTSD do experience inhibition deficits (Aupperle et al., 2012). A recent meta-analysis, which analyzed all EF tasks in a single analysis without distinguishing between EF components found an effect size of *d* = 0.45 (Scott et al., 2015). Importantly, unlike OCD, co-occurring depression may account for EF deficits in individuals with PTSD, although more research in individuals without severe depressive symptoms is needed to confirm this finding (Polak et al., 2012).

There has been little research on EF in anxiety disorders, and that limited literature has yielded mixed findings. While a few studies have found impairments in shifting associated with panic disorder, social anxiety disorder and generalized anxiety disorder (Cohen et al., 1996; Airaksinen et al., 2005; Mantella et al., 2007), others have found no evidence of impairment in shifting (Purcell et al., 1998; Airaksinen et al., 2005; Boldrini et al., 2005), or inhibition (van den Heuvel et al., 2005; Van der Linden et al., 2005; Price and Mohlman, 2007). However, research in non-clinical samples suggests that trait anxiety, and especially anxious apprehension (worry) is associated with impairments in a specific aspect of EF, inhibiting competing responses (Bishop, 2008; Snyder et al., 2010, 2014; Eysenck and Derakshan, 2011). There has been very little research on WM in individuals with anxiety disorders, but there have been reports of impaired visuospatial WM in individuals with panic disorder (Boldrini et al., 2005), and impaired verbal WM manipulation, but not maintenance, in individuals with generalized anxiety disorder (Christopher and MacDonald, 2005). In addition, there is evidence that poor EF might contribute to attentional bias toward threat in anxious individuals, which in turn is involved in maintenance of anxiety (Heeren et al., 2013). A full discussion of the extensive attention bias literature is beyond the scope of this review, and we refer interested readers to recent comprehensive reviews of this topic (Bar-Haim et al., 2007; Heeren et al., 2013).

Attention deficit hyperactivity disorder (ADHD) in both children and adults is associated with impairments in shifting, inhibition, visuospatial WM and verbal WM manipulation, with small-to-medium effect sizes, while verbal WM maintenance is much less impaired (small effect size). Updating has not been widely studied in individuals with ADHD. While earlier theories posited a core inhibitory deficit that secondarily disrupts other aspects of EF (e.g., Barkley, 1997), recent meta-analyses demonstrate that only motor response inhibition tasks (e.g., stop signal and go/no-go) show substantial deficits, while the Stroop measure of inhibition shows only a small effect size. EF is also impaired in other externalizing disorders, including oppositional defiant disorder and conduct disorder, but these deficits may be accounted for at least in part by co-occurring ADHD (see Ogilvie et al., 2011 for meta-analysis).

Finally, there is evidence of deficits in EF associated with substance use disorders. Meta-analyses suggest that there are deficits in response inhibition associated with use/dependence on cocaine, MDMA, methamphetamine, tobacco, and alcohol, but not opioids or cannabis (Smith et al., 2014), and deficits in shifting and inhibition associated with cocaine use (Spronk et al., 2013). Another review found shifting, inhibition, and WM impairments across most substance use disorders, generally with medium effect sizes, but did not perform a formal meta-analysis (Fernandez-Serrano et al., 2011). However, a meta-analysis found no deficits on a composite of inhibition and shifting tasks for chronic opioid users (Baldacchino et al., 2012), only a very small effect size for MDMA use (EF composite *d* = 0.25, Zakzanis et al., 2007). There is evidence that EF deficits persist in medium term abstinence for cocaine (EF composite *d* = 0.32, WM composite *d =*0.52; Potvin et al., 2014) and alcohol dependence (EF composite *d* = 0.57, WM composite *d* = 0.49; Stavro et al., 2012). These meta-analytic reviews on EF impairments in substance use are particularly difficult to interpret. First, the inclusion of poly-substance users makes effects of individual drugs of abuse difficult to isolate. Second, given the neurotoxic effects of alcohol and many drugs of abuse, it is unclear to what extent EF deficits are a cause or consequence of substance use. A recent review of the limited number of longitudinal studies of adolescent heavy drinkers suggests that poor EF is likely both a risk factor and consequence of heavy drinking in adolescents (Peeters et al., 2014). There is also some longitudinal evidence that poor EF may be a risk factor for other substance use disorders (e.g., Nigg et al., 2006), but much more research is needed on this topic.

### Complex Tasks: Verbal Fluency and Planning

Many complex tasks may tap multiple aspects of EF. For example, verbal fluency tasks (generating words starting with a certain letter or from a category) likely tap several cognitive processes (e.g., Rende et al., 2002). Planning tasks are also complex, involving multiple cognitive demands (Goel and Grafman, 1995), and so may not represent a single EF ability. This is problematic if the goal is to understand which specific EF processes are impaired, an issue we return to in the Methodological Issues section. These tasks have nonetheless been frequently used in studies of EF in clinical populations.

Deficits in verbal fluency are widespread across disorders. Indeed, meta-analyses show that out of all the EF tasks included in meta-analyses, the largest deficit for adults with schizophrenia and depression is found on the semantic verbal fluency task, with large and medium effect sizes respectively (**Table 2**). Semantic verbal fluency is also impaired in individuals with BD (medium effect sizes), OCD (small effect sizes), and ADHD (small effect sizes), while there is inconsistent evidence for verbal fluency in individuals with PTSD (Aupperle et al., 2012). For schizophrenia, BD, and MDD, effect sizes for phonemic verbal fluency are somewhat smaller than those for semantic, although still significant. In contrast, individuals with OCD have equal impairments in the two forms of verbal fluency, and verbal fluency deficits associated with ADHD appear to be larger for phonemic verbal fluency than semantic verbal fluency. There has been little research on verbal fluency in anxiety disorders: one study reported impaired phonemic verbal fluency in individuals with panic disorder (Gladsjo et al., 1998), while others found no impairment in individuals with generalized anxiety disorder (Airaksinen et al., 2005) or social anxiety disorder (Hood et al., 2010). However, conclusions are premature given the paucity of evidence.

Why might semantic and phonemic verbal fluency tasks be differentially affected in different disorders? Verbal fluency tasks impose multiple EF demands (e.g., shifting among subcategories, monitoring for repeated words, memory retrieval). One possibility as to why semantic verbal fluency is more impaired than phonemic verbal fluency in individuals with schizophrenia, BD, and depression is that it may place heavier demands on shifting, and particularly on switching between subcategories in a self-directed manner (Snyder and Munakata, 2010, 2013). For example, an individual who has difficulty switching between subcategories might name five farm animals when naming animals, and then fruitlessly continue to try to think of additional farm animals rather than switching to pets or zoo animals. Another possibility is that deficits in semantic memory retrieval may contribute to semantic verbal fluency impairment, particularly in individuals with schizophrenia. For example, a meta-analysis found that individuals with schizophrenia have large deficits on semantic verbal fluency both for switching between subcategories, an index of EF, and semantic clustering, an index of semantic memory (Doughty and Done, 2009). In contrast, the larger effect for phonemic verbal fluency in individuals with ADHD could potentially be due to deficits in phonological processing in many individuals with ADHD, since ADHD and reading disabilities frequently co-occur (Willcutt et al., 2007). Thus, deficits in verbal fluency may arise from a variety of sources, and illustrate the difficulty of interpreting results from complex tasks.

Planning has been much less studied than verbal fluency. Individuals with depression and BD have significant impairments in planning (**Table 2**). In individuals with ADHD, two meta-analyses found quite different effect sizes for planning tasks, one small, one medium. Likewise, meta-analyses of individuals with OCD found different effect sizes for planning, two small and one medium (**Table 2**). Finally, there is inconsistent evidence for planning deficits associated with PTSD (Aupperle et al., 2012). Thus, while planning tasks in theory tap multiple aspects of EF, standard measures of planning may be less sensitive than other EF tasks in detecting more subtle EF deficits associated with some disorders.

### Summary of Previous Findings

In sum, it is clear that the preponderance of evidence shows that deficits on a wide variety of EF tasks are associated, at least concurrently, with many prevalent psychopathologies. Most disorders are associated with fairly uniform deficits EF tasks, although there are some notable variations in effect sizes (e.g., larger deficits for updating than other aspects of EF in OCD, and larger deficits for motor response inhibition than Stroop for ADHD). Thus, the results appear to be consistent with broad, and transdiagnostic, impairment in EF. The exception is simple verbal WM maintenance, which shows smaller, and in for some disorders non-significant, deficits. The finding that manipulation is more impaired than maintenance, along with evidence that visuospatial and verbal WM manipulation are equally impaired, both support the view that WM deficits in these disorders are due to impairment in the central executive aspect of WM, rather than the content-specific maintenance systems (Barch, 2005), again consistent with the view that there are broad impairments in EF associated with psychopathology, rather than impairments in a few individual specific aspects of EF.

Given the wealth of evidence already collected, at this point and with the current state of knowledge, the field generally does not need more cross-sectional, case-control designs comparing a group with one specific disorder to healthy controls on individual standard neuropsychological EF tasks. Such studies only address the question of whether there is a difference in EF task performance between groups, and that question has been satisfactorily answered in the affirmative, at least for disorders and tasks reviewed here (with the exception of the less-studied anxiety disorders). Rather, there is now the opportunity to build on the foundation of such previous studies to better understand the specific mechanisms and causal processes contributing to EF deficits in psychopathology, and to move toward translational applications.

## Limitation of Previous Research and Suggestions for Future Research

Executive function is a challenging topic to study – it is both elusive to define (e.g., Jurado and Rosselli, 2007) and difficult to measure. Critically, interpretation of both the primary literature and meta-analyses is limited because tasks classified as tapping a particular aspect of EF may not be sensitive measures of that process, and are not pure measures of that EF process, since they often require other EF processes as well as multiple non-EF processes. Here we outline limitations in how EF has been defined, conceptualized and measured in previous research with clinical populations, and we present concrete suggestions for addressing these limitations in future research. This selective review is intended to survey the fundamentals of current models of EF, and best practices for assessing EF, for a clinical scientific audience; reviews and resources on specific topics are referenced throughout for those desiring more in-depth information on these topics.

### Conceptual Issues: Models of EF

Many previous studies of EF in clinical populations have either treated EF as unitary, or conversely as a long list of separate, specific abilities. The first of these approaches *over-lumps* diverse tasks into a single construct, for example drawing conclusions about EF in general on the basis of single tasks, which differ from study to study. The second approach *over-splits*, treating a laundry list of tasks, such as decision making, planning and verbal fluency tasks, as if they were assessing separate abilities rather than a common set of component processes that support completion of these more complex tasks.

Rather than showing only “unity” or “diversity” as these two approaches imply, the best current evidence indicates that individual differences in EFs show *both* unity and diversity, an idea originally proposed by Teuber (1972). That is, different components of EF correlate with one another, thus tapping some common underlying ability (unity), but they also show some separability (diversity). This general structure of both common and specific elements of EF is shared by several different models of EF (e.g., Duncan et al., 1997; Baddeley and Repovs, 2006), although different models have focused on different components of EF, and different levels of analysis (e.g., behavioral vs. neural). At the behavioral level of analysis, different prominent models have focused on partly overlapping sets of EF components. For example, Baddeley (1996, 2012) has proposed a central executive system containing subsystems for coordinating performance between tasks, focusing/resolving interference from distractors, switching between tasks, and interfacing with long term memory, while Diamond (2013) has proposed inhibition, WM and cognitive flexibility as core aspects of EF. Others have proposed two-factor models of EF, including top–down modulation of lower-level processes and monitoring processes (Shallice, 2002) or maintenance of task goals in WM and resolution of response competition (Engle and Kane, 2003). At the neural level, models have proposed that distinct but interconnected prefrontal regions support functions such as setting task goals, initiating responses and monitoring performance (Stuss and Alexander, 2007; Stuss, 2011), or using task goals to modulate lower-level processes, resolving competition and evaluating responses (Banich, 2009). It is important to note that while these different models differ in some important ways, they also have many points of convergence, and in many cases largely agree on the core cognitive and neural mechanisms involved in EF despite frequently employing different terms for those processes. These different models and components of EF can all be important to consider in addressing different research questions.

**FIGURE 1:**
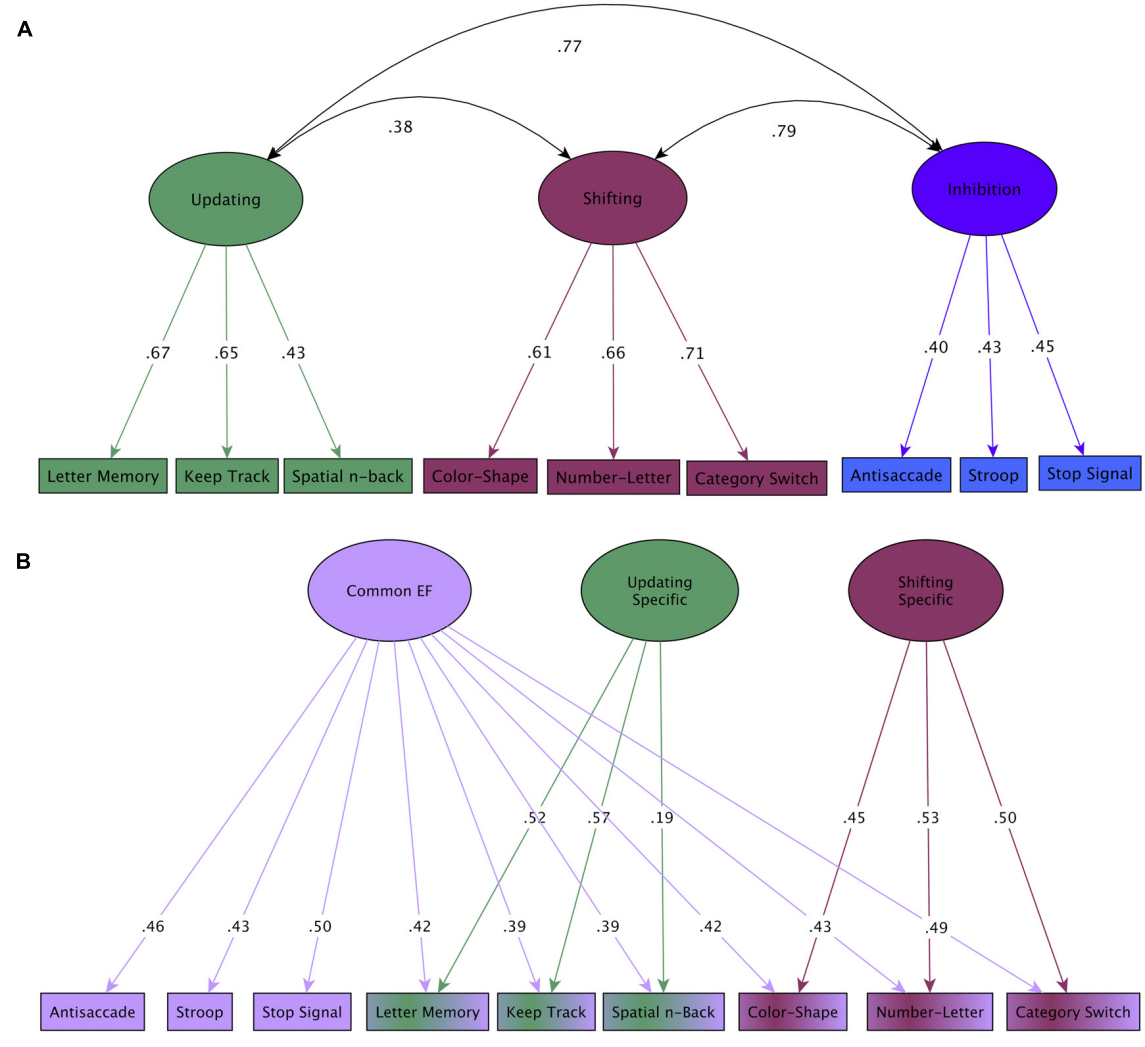
**Unity/diversity model of EF.** Two complimentary ways of representing the unity/diversity model (Adapted from Friedman et al., 2011). In both latent variable models, individual tasks are combined to form latent factors. Numbers on arrows are standardized factor loadings (range 1 to -1), that indicate the extent to which each task is predicted by the latent factor. Those on curved double-headed arrows are correlations between the latent variables, which indicate how strongly they are related. **(A)** The updating, shifting, and inhibition components are substantially correlated (unity), but are separable (i.e., not correlated 1.0; diversity). **(B)** Unity and diversity are more clearly shown with a bifactor model. All nine tasks load onto a common EF factor (unity), and updating and shifting tasks also load onto their respective specific factors (diversity). Note that there is no inhibition-specific factor (i.e., inhibition task variance is fully accounted for by common EF).

Here we focus on one such model, the *unity/diversity model* (Miyake et al., 2000; Friedman et al., 2008; Miyake and Friedman, 2012)*,* because it captures several features of what we believe to be the key components of EF, is practical to use for understanding EF at the behavioral level (e.g., as opposed to models at the neural level which require neuroimaging evidence), and has the potential to shed light on commonalities and differences in EF impairments across clinical populations by differentiating common and specific components of EF. The unity/diversity model focuses on three aspects of EF: (i) updating WM, (ii) shifting, and (iii) inhibition, as well as a common EF ability which spans these components. There are substantial but far from perfect (i.e., 1.0) correlations among shifting, updating and inhibition factors (Friedman et al., 2011), illustrated here in a large sample of 17 years old twins (**Figure 1A**). This general unity/diversity pattern has been consistently found in other samples, including children (e.g., Lehto et al., 2003; Rose et al., 2011), young adults (Miyake et al., 2000), and older adults (e.g., Vaughan and Giovanello, 2010), although only a single unitary factor (e.g., Wiebe et al., 2008, 2011) or two factors (Miller et al., 2012a) may be evident in preschool children. Each EF ability (e.g., updating) can be decomposed into what is common across all three EFs, or unity (common EF), and what is unique to that particular ability, or diversity (e.g., updating-specific ability; **Figure 1B**).

Thus, in order to specify the cognitive and biological underpinnings of EF, the unity/diversity framework suggests it is necessary to decompose task performance into common (common EF) and specific (updating- specific, and shifting-specific) abilities that may more cleanly map onto the underlying cognitive processes. This approach is relatively new, but has already produced some important discoveries. First, after accounting for common EF, there is no unique variance left for inhibition (i.e., no inhibition-specific factor), a finding that has been replicated in two independent samples (Friedman et al., 2008, 2011) – that is, individual differences in common EF fully account for individual differences in inhibition (**Figure 1B**). This finding is consistent with the view that the ability thought to be captured by common EF-actively maintaining task goals and goal-related information and using this information to effectively bias lower-level processing – is the key EF requirement of response inhibition (whereas stopping itself may be relatively automatic; (e.g., Munakata et al., 2011; Chatham et al., 2012), but see (e.g., Aron, 2007; Diamond, 2013) for alternative views of inhibition).

Second, common EF and shifting-specific components sometimes show opposing patterns of correlations with other measures, consistent with hypothesized trade-offs between stability (common EF) and flexibility (shifting-specific) suggested in the literature (e.g., Goschke, 2000). For example, rumination is associated with better performance on an EF task requiring goal maintenance (stability) but worse performance on an EF task requiring rapid shifting (flexibility; Altamirano et al., 2010), and young children who show good self-restraint (not reaching for an attractive toy they have been told not to touch) go on to have higher levels of common EF (stability) but lower levels of shifting-specific EF (flexibility) as adolescents (Friedman et al., 2011). Critically, these findings suggest that specific deficits in stability or flexibility will only be apparent when performance on shifting tasks is decomposed into common EF and shifting-specific factors.

Most importantly for clinical research, the different components of EF identified by the unity/diversity model differentially predict individual differences in clinically important behaviors (Friedman et al., 2007, 2011; Young et al., 2009). In particular, recent evidence points to common EF as the primary source of such predictive power. For example, poor common EF is associated with *behavioral disinhibition,* a general vulnerability factor hypothesized to underlie externalizing behavior problems, such as ADHD, conduct disorder, substance use, and novelty seeking/risk taking (Young et al., 2009). The similarity of effect sizes across the core EF domains in other disorders (**Table 1**) suggests that psychopathology more broadly may be associated with impairment in common EF. While this possibility has not been formally tested for most disorders, it suggests that decomposing EF into its common and specific components may have important implications for understanding EF deficits associated with psychopathology, an issue we return to in Future Directions.

### Methodological Issues

#### Multiple Measures

**FIGURE 2:**
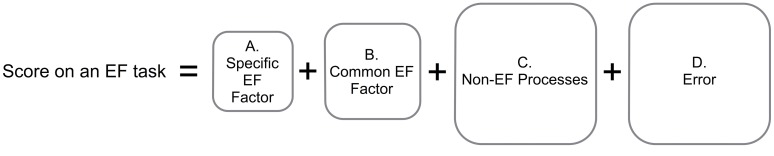
**Task impurity problem.** A score on an EF task is composed of **(A)** systematic variance attributable to the specific aspect of EF targeted by that task (e.g., shifting-specific or updating-specific variance), **(B)** systematic variance attributable to common EF (i.e., variance shared across multiple types of EF tasks, hypothesized to be related to task-goal maintenance), **(C)** systematic variance attributable to non-EF aspects of the task (e.g., articulation speed, visual processing), and **(D)** non-systematic (error) variance. Use of single tasks to measure EF is thus problematic because this task-impurity makes interpreting the results difficult and because the amount of variance attributable to EF **(A,B)** can be relatively small compared to non-EF variance **(C,D)**.

Arguably the most vexing problem in effectively and precisely measuring EF is the task-impurity problem (**Figure 2**). Because any target EF must be embedded within a specific task context (so that the target EF has something to operate on), all EF tasks necessarily include systematic variance attributable to non-EF processes associated with that specific task context (e.g., color processing and articulation speed in the Stroop task, visuospatial processing in a spatial *n*-back task; e.g., Miyake et al., 2000). Unfortunately, this systematic non-EF variance and measurement error (random noise in the data) are substantial, making it difficult to cleanly measure the EF variance of interest (**Figure 2**). In addition, even targeted EF tasks tap both specific and common aspects of EF (e.g., common EF plus updating-specific for updating tasks). Because most studies of EF in clinical populations have used only a single task to assess EF processes of interest, results reported in this literature are nearly always a mixture of non-EF, common EF, and specific EF component effects, making interpretation of the results difficult. For example, poor performance on a spatial *n*-back task could arise from impaired common EF, updating-specific EF, or non-EF spatial processing problems.

This task impurity problem can be alleviated by using multiple measures of each EF component under investigation. In this approach, multiple exemplar tasks are chosen that capture the target ability (e.g., three tasks that require updating WM; Miyake et al., 2000; Friedman et al., 2008) but seem different on the surface (e.g., the nature of the information to be updated is different in each). If exemplar tasks are chosen such that they share little systematic non-EF variance, one can statistically extract what is common across those tasks and use the resulting “purer” latent variable as the measure of EF. We thus suggest that whenever possible, researchers administer multiple tasks that target the specific aspect of EF they have hypotheses about, while also including some additional tasks to demonstrate the specificity of effects (c.f. Goschke, 2014). If researchers are interested in measuring Common EF, measures of each EF component (shifting, updating, and inhibition) should be used, and then aggregated.

Several methods are available for combining data from multiple measures, depending on the sample size of the study. The simplest and most versatile approach is to calculate a *z*-mean across tasks, which can be done with any sample size. When *z*-means across tasks are used in place of individual tasks, variance in the scores not related to the construct of interest (e.g., updating) can no longer drive the effects, so long as it is not systematic across the averaged tasks (e.g., the updating tasks do not share other task requirements in common). The disadvantage of this approach is that it merely combines scores – the error variance is not removed, and can still be a source of reduced power. For this reason, if sample size is large enough, it is preferable to use latent variable approaches for extracting only the variance shared across tasks while removing the error variance, using such multivariate statistical techniques as confirmatory factor analysis and structural equation modeling. While this approach may not be practical when there are a limited number of participants available for study (e.g., for disorders with low prevalence rates) or strict limitations on the amount of time available for testing, it has great potential for testing theories of relations between specific aspects of EF and psychopathology, as discussed further below. If time limitations only permit collecting data on a single task, researchers should be aware of the inherent limitations this imposes on the conclusions that can be drawn given the task impurity problem, and thus suitably cautious in interpreting the results.

#### Task Selection

In addition to using multiple tasks, it is essential to pick tasks carefully. Many studies of EF in clinical populations currently use traditional neuropsychological EF measures that tap multiple aspects of EF as well as non-EF abilities. These tasks may be useful for screening individuals for severe EF deficits, however, they are too broad to answer fine-grained questions about specific aspects of EF that may be implicated in psychopathology. As discussed above, complex neuropsychological tests tap a wide variety of cognitive processes, including not only multiple aspects of EF, but also non-executive abilities, making such measures difficult to interpret. For example, multiple disorders are associated with impairments in verbal fluency, but it is unknown if those impairments arise from deficits in shifting, WM, or non-EF aspects of the tasks, or some mixture of these factors, and if this differs among disorders. These concerns can be addressed by using tasks designed to more specifically place demands on individual aspects of EF (e.g., Miyake et al., 2000; Aron, 2008; Goschke, 2014). These tasks need not necessarily entirely replace traditional neuropsychological tasks, but if they are used it is important to also include more specific tasks in order to identify what specific processes account for impairment on the broader neuropsychological tasks.

In addition, in place of EF tasks, many studies in the clinical literature have used questionnaires to assess self or other (e.g., parent, teacher) report of behaviors putatively related to EF (e.g., Behavioral Rating Inventory for EF, measures of effortful control temperament). However, these questionnaires correlate relatively poorly with task-based measures of EF (Toplak et al., 2012), and thus should not be assumed to be measuring the same constructs. Questionnaire-based measures ask about behavior in complex real-world situations (e.g., completing tasks on time, staying organized). This has advantages in terms of ecological validity, and some have argued in favor of using questionnaires rather than EF tasks (e.g., Barkley and Fischer, 2011). However, questionnaire measures pose interpretational problems even greater than those posed by complex tasks like verbal fluency. That is, these real-world behaviors involve multiple executive and non-executive processes, and can also be heavily influenced by contextual factors – for example, responses to a question about completing homework on time may depend not only on various aspects of EF, but also motivation to do well in school, and whether there is a quiet place to work at home away from distractions, among other factors. Thus, specific questions about EF impairments are best addressed using targeted EF tasks. Questionnaire measures may be valuable to include as a measure of real-world behavior, but should not be interpreted as necessarily reflecting EF *per se*.

In addition to selecting more targeted EF tasks, it is important to consider the sensitivity and reliability of EF tasks. Tasks should be selected that are sensitive to the magnitude of deficits expected for the sample being tested. Many traditional neuropsychological tests (e.g., Trail Making Test, WCST) were originally designed to assess EF deficits in patients with frontal lobe damage or dementia. These tasks may not be sufficiently demanding to be sensitive to more subtle deficits in EF associated with psychopathology (i.e., they may have ceiling effects, with all participants performing well).

The reliability of tasks is also an important issue. Tasks with low reliability necessarily have poor correlations with other measures (e.g., measures of psychopathology). Unfortunately, complex EF tasks tend to have relatively low internal and/or test–retest reliability, potentially because people adopt different strategies at different times when completing the tasks (Miyake et al., 2000). It is also important to note that reliability is sample specific – for example, reliability may be high in severely impaired individuals (e.g., they reliably fail to maintain more than a few items in a WM task) but reliability may be lower in a less impaired sample (e.g., they start out doing poorly but then are able to improve their performance by using a more effective rehearsal strategy).

It is thus important to select the most reliable tasks available, determine the reliability of tasks within the population of interest (e.g., specific diagnostic group, healthy control group, age group, etc.), and plan sample sizes accordingly to achieve adequate power to detect the expected effect sizes given the reliability of the measures. Problems with task sensitivity and reliability are problematic because they may lead to false negative findings that either result in the study not being published (the file drawer problem), or being published with the erroneous conclusion that EF is not impaired in the clinical group (lower effect sizes, and thus problematic for accurate reviews via meta analysis). Indeed, many studies in the clinical literature have argued against the existence of EF deficits in particular populations based on null results that may have arisen from power limitations due to poor task sensitivity and reliability and/or small sample size, rather than reflecting a true lack of impairment.

The cognitive psychology and cognitive neuroscience literatures contain a rich source of targeted and sensitive paradigms, and these can easily be used for clinical studies as well. For example Miyake et al. (2000), Friedman and Miyake (2004) and Friedman et al. (2008) have developed and adapted multiple tasks to assess inhibition, shifting, and updating. Additional more specific EF tasks are listed in the fourth column of **Table 1**. Thus, once researchers determine what aspects of EF they wish to investigate, it should not be too difficult to find established tasks offering much more specificity and construct validity than the traditional, less specific neuropsychological measures frequently used in clinical studies.

There are also a number of commercially available task batteries that include tasks assessing EF (e.g., the Cambridge Neuropsychological Test Automated Battery, CANTAB), as well as some freely available task batteries (e.g., the NIH Toolbox). These batteries have both advantages and disadvantages. On the plus side, these batteries often have more extensive psychometric evaluations and norms, and their standardization allows for clear comparison across studies. However, these batteries generally do not provide comprehensive coverage of different components of EF aligned with current models (e.g., CANTAB has visuospatial WM, planning and shifting tasks, but no tasks assessing other aspects of EF), and do not generally provide multiple measures of each construct needed for latent variable approaches. Moreover, since these test batteries have been heavily used in most clinical populations, further case-control studies with these tasks are unlikely to yield new insights. These advantages and disadvantages should be carefully considered when deciding whether to use a pre-packaged task battery vs. selecting EF tasks from the cognitive psychology/neuroscience literature.

#### Other Methodological Considerations

Of almost equal importance to what tasks are used to evaluate EF is how the data from those tasks are collected and analyzed. When the total individual variance in EF task performance is broken into EF, task-specific and error components, the “noise” of non-EF task-specific variance and error variance can be quite large, while the “signal” of EF-specific variance may be quite small (**Figure 2**). Thus, in order to detect the signal that is of central importance for scientific inquiry, it is critically important to minimize error variance and maximize power. First, there is a strong need to increase sample size to improve power. Many previous studies have been underpowered, which likely leads to a file drawer problem and lack of replicability (e.g., Pashler and Wagenmakers, 2012), and problematically also leads to potentially erroneous claims that there is no EF impairment (e.g., that anxiety does not impair updating, (Eysenck et al., 2007). Second, once the data are collected, the reliability and validity of the measures depend critically on how they are screened and analyzed. For any given task, it is important to use the most specific, sensitive, and reliable measure of task performance. For the suggested more specific EF tasks in **Table 1**, the citation for each task provides a description of how to calculate measures of task performance. It is also important to screen for and appropriately address the presence of outliers, both outlier trials for each participant and outlier participants. Such outliers contribute to error variance and distort results, potentially leading to either false negatives or false positives. Taking these steps to collect and derive the highest quality EF measures possible maximizes the chances of detecting EF deficits and producing valid, interpretable results in clinical science.

## Future Directions

Thus far we have reviewed evidence that multiple forms of psychopathology are associated with impairment on multiple measures of EF, and discussed what we see as the key conceptual and methodological limitations to this previous research. Namely, many previous studies of EF in clinical populations have either treated EF as unitary, or conversely as a long list of separate, specific abilities, counter to the best current evidence indicating that individual differences in EFs show *both* unity and diversity. (Here we focus on one such model, the *unity/diversity model* Miyake et al., 2000; Friedman et al., 2008; Miyake and Friedman, 2012)*,* which we believe may be a particularly useful framework for clinical research, however, the same points largely apply to other models of EF.) In order to apply these current, best supported models of EF to clinical research, it will also be necessary to address a number of methodological limitations of previous research, by using multiple, specific, sensitive, and appropriately analyzed measures of different components of EF. Moreover, the vast majority of previous research has taken the form of cross-sectional case-control studies in adults, which are unable to differentiate between different possible causal links between EF and psychopathology (e.g., cause, consequence, or correlate).

Given these limitations to previous research and the goal of understanding links between EF and psychopathology at a level of detail and specificity that can support translational research, we propose two broad directions for future research. First, we suggest that the problem of understanding the seemingly undifferentiated nature of EF impairments across disorders may be made more tractable by testing models that include both unity and diversity, in both psychopathology and EF. Second, we suggest that research will need to move beyond cross-sectional case-control designs to test different possible causal links between EF and psychopathology.

### Testing Models of Unity/Diversity Across Both EF and Psychopathology

What gives rise to broad patterns of impairment in EF across most disorders? First, these deficits cannot be easily explained by non-specific factors such as psychomotor slowing, differences in IQ or education, or medication use (e.g., Barch, 2005; Forbes et al., 2009; Snyder, 2013; Snyder et al., 2015). Second, in most cases, effect sizes are similar across the core EF domains. This pattern of broad impairment across most EF tasks found by meta-analyses is consistent with the theory that individuals with multiple forms of psychopathology have impairments in the unitary component of EF (i.e., common EF), posited to be the ability to actively maintain task goals and use this information to provide top–down support for task-relevant responses (Friedman et al., 2008; Miyake and Friedman, 2012).

We view this theory as fully compatible with others who have posited impairment in “executive attention” associated with psychopathology (e.g., Gooding et al., 2006; Pacheco-Unguetti et al., 2010; Orellana et al., 2012; Maurage et al., 2014). First, conceptually, in the dominant model of attention, the executive attention network is defined as similarly to common EF, as involving task set maintenance to provide top–down control supporting resolution of competition between response options (Posner and Petersen, 1990; Petersen and Posner, 2012). Second, empirically, this executive attention process is predominantly assessed with the flanker interference component of the Attentional Networks Task (ANT, Fan et al., 2002), and flanker task interference is strongly correlated with prepotent response inhibition at the latent level (Friedman and Miyake, 2004), which in turn is fully accounted for by common EF (e.g., Friedman et al., 2008). Finally, other attentional processes (alerting and orienting) appear to be largely unimpaired in individuals with psychopathology e.g., Gooding et al., 2006; Pacheco-Unguetti et al., 2010; Orellana et al., 2012; Maurage et al., 2014), suggesting that deficits are not due to lower-level attentional difficulties. Thus, we argue that the finding of impairments on the executive attention component of the ANT is fully compatible with impairment in Common EF. Although other explanations are also possible (e.g., multiple specific aspects of EF could be independently impaired), impairment in common EF is the most parsimonious interpretation.

Importantly, psychopathology has also been shown to consist of both common and specific factors. Specifically, latent variable models of psychopathology in both adolescents and adults find that there is a common factor that spans all aspects of common psychopathologies, in addition to factors for more specific aspects of psychopathology (internalizing and externalizing; e.g., Lahey et al., 2012; Tackett et al., 2013; Caspi et al., 2014). This general psychopathology factor, recently dubbed the “p Factor,” is related to broad negative emotionality (neuroticism) and associated with low conscientiousness and agreeableness, and more life impairment (Caspi et al., 2014).

This raises the possibility that broad, transdiagnostic impairments in EF might be explained by a link between this p Factor and common EF. Indeed, the p Factor has been shown to be associated with poorer performance on cognitive tasks including EF tasks, indicators of poor cerebrovascular functioning, and self-reported cognitive and self-control problems, assessed as early as 3 years of age (Caspi et al., 2014). These findings suggest that early neurological and cognitive problems may be a general liability factor for psychopathology. However, the nature of these cognitive problems has not yet been conclusively tested. Future research could test the hypothesis that these seemingly broad cognitive problems associated with common psychopathology are best explained as a deficit in common EF.

It is also possible that individuals with psychopathology have processing-specific impairments in shifting and/or updating (recall that there is no inhibition-specific component, e.g., Friedman et al., 2008) in addition to deficits in common EF, which could either be associated with common psychopathology (p Factor), or more specific aspects of psychopathology (e.g., depression, OCD, ADHD, etc.). Examining links between both common and specific aspects of EF and psychopathology has the potential to greatly clarify the nature of EF impairments associated with particular forms of psychopathology, and thus accelerate progress in understanding how EF impairments may contribute to both comorbidity across disorders and heterogeneity within disorders (e.g., anhedonia vs. broad negative affect in depression, anxious arousal vs. anxious apprehension in anxiety disorders, etc.).

While examining common EF as a potential transdiagnositic risk factor for common psychopathology is a highly promising direction for future research, it is important to bear in mind that cognitive factors that appear transdiagnostic at one level of analysis may not be when more detailed measures at multiple levels of analysis are considered. Just as many problems with a car (e.g., a dead battery, broken starter, or being out of gas) could all lead to the same outcome (the car won’t start), the same cognitive endpoint might be reached by many different underlying mechanisms (*equifinality*). Thus, while EF deficits appear to be a transdiagnostic feature of psychopathology at the level of performance on neuropsychological tasks, in some cases these shared behavioral deficits may arise from distinct neural mechanisms (e.g., different perturbations in neurotransmitter systems, (e.g., Gigante et al., 2012; Luykx et al., 2012). Thus, determining whether a product or process is truly transdiagnostic requires escaping both diagnostic and methodological silos to consider underlying mechanisms at multiple levels of analysis.

### Causal Models

Though there are notable exceptions, a general shortcoming of the broad field of cognitive risks in psychopathology across the lifespan is the frequent lack of consideration of possible models of how cognitive impairments and psychopathology may be causally related. Specifically, it is unknown if EF deficits (a) precede, and are a potential causal risk factor for, developing psychopathology, (b) follow, and are a consequence of psychopathology, (c) are a correlate of psychopathology without playing a causal role (e.g., both poor EF and psychopathology may be related to a third factor), or some combination of these models (e.g., transactional models; c.f. Goschke, 2014). It is important to note that these logical models are not mutually exclusive. Indeed, it is highly likely that different models will hold true for different forms of psychopathology or aspects of EF, or even for the same disorder and cognitive process at different times or for different individuals.

While many studies explicitly or implicitly assume a particular causal model, there have been far fewer attempts to try to rule out, or in, particular models based on the evidence. Critically, cross-sectional case-control studies are not capable of differentiating between these possible models. While there is still a place for cross-sectional research in clarifying the nature of EF deficits in different clinical populations (e.g., using latent variable models to examine common vs. specific deficits), an important next step will be to build on these cross-sectional results with longitudinal, neural, and behavior genetic studies that can be informative in testing putative causal models.

For example, a small number of prospective longitudinal studies have been conducted, and suggest that impairments in EF and related brain systems predict later psychosis, ADHD, and PTSD, suggesting that cognitive deficits may be a risk factor for many disorders (Cannon et al., 2006; Parslow and Jorm, 2007; Campbell and Stauffenberg, 2008). Moreover, there is some evidence that EF deficits are primarily state-independent (manifest even when illness symptoms are not present, (e.g., Kurtz and Gerraty, 2009; Snyder, 2013) and present in attenuated form in unaffected family members of individuals with schizophrenia, BD, OCD, and PTSD, suggesting EF deficits may be an endophenotype for many forms of psychopathology (e.g., Barch, 2005; Gilbertson et al., 2006; Menzies et al., 2007; Bora et al., 2009). While these data showing associations between premorbid EF or genetic factors and EF impairments associated with psychopathology are consistent with causal risk factor or endophenotype models, in other cases there is evidence supporting the consequence model. For example, a meta-analysis found progressive loss of gray matter in the PFC and temporal lobe in individuals with schizophrenia, especially during the first episode, suggesting that the onset of schizophrenia triggers a neurodegenerative process that could impair EF (Vita et al., 2012).

In sum, the causal links between EF and psychopathology have not been well established, and the cascade of mechanisms connecting EF to psychopathology are unknown and in need of theoretical and empirical investigation. Besides being of importance for basic research, these questions have important implications for prevention and treatment. For example, if EF deficits are a risk factor for psychopathology, individuals who are vulnerable to, but have not yet developed, psychopathology (e.g., due to parental history) might benefit from early intervention to teach compensatory strategies to mitigate the effects of EF impairments, a topic we expand on next.

### Treatment Implications

Better understanding EF deficits associated with psychopathology has important implications for evidence-based assessment and intervention advancement, including enhancing screening, prevention, and treatment and better understanding treatment mechanisms. In terms of prevention and treatment approaches, current evidence suggests that approaches aimed at teaching compensatory strategies may be the most promising direction for future translational research. Importantly, there is little evidence in support of direct training of EF (i.e., targeting the weakness rather than compensatory strategies). In general, the majority of studies have found that while task performance improves, there is little evidence that training effects generalize to real-world function or improve clinical symptoms (e.g., for review see Rabipour and Raz, 2012). That is, these interventions appear to improve the task-specific non-EF processes (**Figure 2C**) but not the EF *per se* (**Figures 2A,B**). One possible exception is EF training in children with ADHD, which some studies indicate training can improve performance on untrained EF tasks, and in some cases parent report of symptoms (Rabipour and Raz, 2012). However, a recent meta-analysis found that EF training did not reliably transfer to academic performance and blinded subjective ratings of children with ADHD (i.e., ratings by individuals who did not know about the training intervention), although there were very small but significant improvements on non-trained cognitive tasks (Rapport et al., 2013). Thus, the jury remains out on possible benefits of direct EF training, but the majority of existing evidence does not indicate effective transfer to improved daily functioning or symptom reduction.

It is not clear to what extent these findings reflect genuine limitations of cognitive training in general, vs. problems with the specific training programs (many of them commercial products). For example, many of the programs focus on training the least impaired aspect of EF in children with ADHD, simple WM maintenance (Rapport et al., 2013). This leaves open the possibility that types of training that better target areas of weakness might provide better transfer. Intriguing findings suggest that certain types of EF training may change the underlying neural mechanisms to be more efficient rather than changing strategy use only (Owens et al., 2013), suggesting that such training might transfer more broadly to processes involving the same neural mechanisms, although this has not yet been tested. As an alternative to training, there is evidence that directly manipulating prefrontal function through non-invasive brain stimulation techniques (repetitive transcranial stimulation and transcranial direct current stimulation) can produce short-term improvements in performance on EF tasks (Brunoni and Vanderhasselt, 2014). However, evidence for efficacy of these techniques in producing long-term improvements in cognition in clinical groups is currently promising but inconclusive (e.g., Demirtas-Tatlidede et al., 2013).

Given the current lack of evidence for effective transfer of EF training, treatment, and prevention programs involving compensatory strategies may be a more promising direction for translational research. For example, goal management techniques (e.g., Goal Management Training; (e.g., Cicerone et al., 2006) may help individuals compensate for poor Common EF by teaching them to break goals into manageable sub-goals and monitor their progress. While these types of compensatory training have most frequently been used with individuals who have sustained brain damage, there is emerging evidence that they may be helpful for individuals with psychopathology as well. Cognitive rehabilitation interventions aimed at teaching compensatory strategies (e.g., use of lists and cues, dividing tasks into smaller steps, etc.), have been shown to improve functional outcomes (e.g., occupational/academic functioning) in individuals with schizophrenia (for review see Kluwe-Schiavon et al., 2013), BD (e.g., Deckersbach et al., 2010), and ADHD (e.g., Hahn-Markowitz et al., 2011). There is less research on cognitive remediation in individuals with depression or anxiety disorders, although it is intriguingly suggestive that some therapies (e.g., behavioral activation; (e.g., Dimidjian et al., 2011) incorporate compensatory strategies (e.g., cues to engage in an activity, like putting walking shoes by the door).

In addition to augmenting treatment with compensatory strategy training, there may be a need to adapt and personalize current treatment approaches to match clients’ EF abilities. Better understanding the EF profile of each patient may be helpful in tailoring treatment approaches. There is some preliminary evidence that pre-treatment EF predicts treatment response to CBT (e.g., Mohlman and Gorman, 2005), potentially because EF is needed to engage effectively with many treatment and prevention strategies. For example, individuals in CBT are asked to do thought restructuring exercises, formulate and implement behavioral plans, and monitor their own cognition and behavior, all of which involve EF (e.g., Mohlman and Gorman, 2005). Thus, identifying specific aspects of EF associated with psychopathology will be critical for determining who is most likely to benefit from existing interventions and who needs adaptations to those interventions or new, and different, interventions, such as those for whom CBT (or other high EF interventions) may not be as efficacious (i.e., personalization of intervention, or what works for whom). In many cases, adapting current interventions to be more manageable for individuals with poor EF may simply be a matter of providing additional support and structure. For example, knowing that an individual has reduced ability to select among multiple competing options might suggest personalization by reducing the number of options offered in the course of therapy (e.g., have the depressed patient choose from only 2–3 behavioral activation options to improve mood instead of choosing from an overwhelming menu of 36 pleasant activities).

Executive function deficits also have important implications for psychopharmacological treatments. First, as for behavioral therapies, pre-treatment EF has been shown to predict drug treatment response. In particular, EF predicts pharmacotherapy response in individuals with depression (e.g., McLennan and Mathias, 2010), schizophrenia (e.g., Kim et al., 2008), OCD (e.g., D’Alcante et al., 2012), and BD (e.g., Gruber et al., 2008). Although the precise reasons are unclear (e.g., there could be neurobiological explanations), poor medication compliance is the most likely and parsimonious explanation because these findings hold across several different types of psychiatric medications. Thus, individuals with poor EF may benefit from additional supports for successful medication management (e.g., pill boxes that sound an alarm when it is time to take medication).

Second, better understanding pathophysiology of EF deficits associated with psychopathology may lead to improved targeting of drug treatments to enhance EF. Currently, there are few medications directly aimed at improving EF, with the exception of stimulant medications for ADHD. The majority, but not all, studies find that stimulant medications improve EF performance in individuals with ADHD (for review see Pietrzak et al., 2006). There are also interesting suggestions that certain medications (e.g., Modafinil, a cognitive enhancer) have potential for improving outcomes in individuals with depression [e.g., improved response to antidepressant treatment with Modafinil, (Abolfazli et al., 2011)], but these effects are not yet well established with EF (Murrough et al., 2011).

Better understanding the specific EF deficits associated with different forms of psychopathology could enhance targeting of medications that affect the neurotransmitter systems known to be involved in those EF processes. For example, GABA and glutamate have been implicated in specific EF processes (e.g., Krystal et al., 2001; Castner and Williams, 2007; Snyder et al., 2010; de la Vega et al., 2014), and these neurotransmitter systems are known to be affected in depression and anxiety disorders (for review see Möhler, 2012; Tokita et al., 2012). Already, promising GABA and glutamate medications exist, or are under development and testing, for anxiety and depression (Möhler, 2012; Krystal et al., 2014). Findings from studies of EF at multiple levels of measurement can inform continued drug development and personalization by identifying the current drugs that better target the most critical pathophysiological processes to maximize efficacy.

Finally, measuring EF over the course of treatment may help identify treatment mechanisms, which in turn can lead to refinements to treatment approaches to better target those mechanisms. For example, there is some emerging evidence that mindfulness interventions increase cognitive flexibility (for review see Chiesa et al., 2011), which may partly mediate positive effects of the intervention on some outcomes (Heeren et al., 2009). However, this hypothesis has not been tested with latent variable approaches that allow flexibility (i.e., shifting-specific EF) to be differentiated from common EF and non-EF aspects of the tasks, an important area for future research. There are many other potential mechanisms of action involving EF that are conceptually plausible but untested, for example, that some positive effects of behavioral activation approaches could be partially mediated by improved goal maintenance. Identifying such mechanisms of action has been noted as an important step in improving treatment efficacy and advancing evidenced-based psychological interventions (e.g., Kazdin, 2007; Emmelkamp et al., 2014).

## Conclusion

In recent decades, a proliferation of research has investigated EF in clinical populations, and for good reason: individual differences in EF are associated with many important aspects of human health and functioning, including most forms of psychopathology. However, despite the strongly interdisciplinary nature of this topic, poised between clinical and cognitive science, these fields have followed largely independent paths. Here we have argued that it will be necessary to move past this model of ‘parallel play’ in order to push clinical psychological science forward toward a better understanding of how and why EF is so broadly compromised across mental health disorders. Specifically, we advocate for better assessment of EF using the best current, validated models of EF and best methods for assessing EF. Critically, interpretation of both the primary literature and meta-analyses to date is limited because typical methods of assessing EF in the clinical literature often lack specificity and sensitivity to the particular aspect of EF they are intended to measure.

To address these limitations, we provided recommendations for applying validated models of EF to clinical research, using multiple tasks to obtain purer measures of EF, and selecting and analyzing tasks in ways that minimize the inherent noisiness of EF data. Specifically, to address the task impurity problem and improve reliability, we recommend carefully choosing EF components to focus on, based on theory and/or past research, and using multiple measures of each EF component of interest and combining them using composite scores or latent variable analysis. When possible, investigating both common and specific components of EF and psychopathology using latent variable approaches holds great promise for making the problem of understanding the seemingly undifferentiated nature of EF impairments across disorders more tractable. We also urge researchers to consider using more specific EF measures (see **Table 1**), instead of or in addition to, traditional, but overly broad, neuropsychological tests. Given the inherent noisiness of even the best EF tasks, it is also critically important to ensure studies are adequately powered, calculate the most specific, sensitive and reliable measure possible from each task (recognizing that this may differ from what is typically reported for traditional neuropsychological tasks; see **Table 1** for method citations), screen for outliers and trim data appropriately both within and across subjects. Taken together, we hope these suggestions for combining the best current theoretical and methodological advances of clinical and cognitive science can help to advance the field toward understanding the underlying mechanisms involved in EF impairments at a level that can enable translational research to improve treatment.

## Conflict of Interest Statement

The authors declare that the research was conducted in the absence of any commercial or financial relationships that could be construed as a potential conflict of interest.
